# Breastfeeding during the COVID-19 pandemic

**DOI:** 10.18332/ejm/123868

**Published:** 2020-08-04

**Authors:** Z. Burcu Yurtsal

**Affiliations:** 1 Midwifery Department, Faculty of Health Science, Sivas Cumhuriyet University, Sivas, Turkey

**Keywords:** breastfeeding, COVID-19, pandemic, midwives

**Dear Editor,**

WHO recommends that newborns be given breast milk only for the first 6 months and that they should continue breastfeeding to two years and over with complementary foods afterwards^1^. COVID-19 originated in Wuhan, China in December 2019 and unfortunately has spread all over the world to become a pandemic. The International Council of Midwives (ICM) expressed concerns regarding the inappropriate protocols management of the human rights of women in childbirth and breastfeeding in perinatal period during the pandemic^2^. There is no current evidence showing that specific symptoms appear in the prenatal, natal and postpartum period and are transmitted from mother to fetus or newborn^2-5^.

WHO, UNICEF, WABA, ILCA’s current recommendation is to continue breastfeeding even with the mother suspected or confirmed to have COVID-19. As before the pandemic, breastfeeding and skin contact are recommended to start immediately after birth. In order to protect and increase the health of the mother and baby, we should encourage and support breastfeeding as before, because we know that breast milk strengthens the immune system of the newborn and protects against some infectious diseases^3-5^.

Breastfeeding, in general, during infection outbreaks should not be interrupted. Mothers who intend to wean their baby or toddler from the breast should postpone such decisions during this special period, to enable the baby or toddler to benefit from the immune components in breast milk^2,4-8^.

It is important to remember that during the outbreak, for various reasons, such as the mother’s COVID-19 infection, deprivation of the baby from breast milk can put it at a greater risk. Breast-fed babies will be vulnerable to all infectious diseases, especially COVID-19, without the protective proteins that the mother’s body actively produces against COVID-19 during the illness and which pass into breast milk and the immune substances naturally found in breast milk^4-8^.

Breastfeeding should not be interrupted in the presence of a contact history of the breastfeeding mother with a person diagnosed with COVID-19 infection. She should continue breastfeeding by taking the necessary precautions, which are: 1) mother wearing a mask while breastfeeding or milking; 2) effective washing of the hands for 20 seconds before breastfeeding; 3) frequent ventilation of the environment; 4) washing clothes at 60–90°C with normal detergent; and 5) drinking plenty of fluids, balanced diet and regular sleep^4-8^. COVID-19 (+) breastfeeding mother should be treated at home. WHO advise that breastfeeding should be continued, paying attention to hygiene rules instead of separating the mother and baby^4-8^.

Midwives are primary responsible health personnel to protect and promote the health of the mother, baby and society, as before the pandemic. In this pandemic process, like other healthcare professionals, midwives work in the frontline to protect and improve maternal, infant and community health. Midwives will continue to support breastfeeding and initiation of breastfeeding after birth as always and everywhere^1-4^.

Maternal care professionals support mother and newborn best, but maternity care professionals also need to be supported. In this pandemic process, the European Midwives Association (EMA) emphasizes that national and EU officials guarantee protection and ensure acceptable working conditions for midwives, so that they can focus on safer and respectful family centered care now more than ever before^9^. Also, midwives, as key professionals in understanding the healthcare and complexities of women in COVID-19, exist to provide a theoretical assessment of ‘medicalized terminology’ and supportive philosophy^10^.

Consequently, there is no obstacle to breastfeeding in the presence of confirmed or suspected COVID-19 infection. The initiation of breastfeeding and its continuation to protect the health of the babies and the mothers is important not only during normal times but also during a pandemic.
